# Conversion Total Knee Arthroplasty After Tibial Plateau Fixation Is Associated With Lower Reimbursement, Greater Complication Rates, and Similar Opioid Use

**DOI:** 10.7759/cureus.25171

**Published:** 2022-05-20

**Authors:** Jacob Wood, Varatharaj Mounasamy, Dane Wukich, Senthil Sambandam

**Affiliations:** 1 Orthopedics, University of Texas Southwestern Medical Center, Dallas, USA

**Keywords:** conversion tka, opioid, cost, complications, tibial plateau

## Abstract

Objective

Total knee replacement after previous open reduction and internal fixation for tibial plateau fracture (conversion total knee) increases the complexity of the procedure and the complication rate. However, very little research exists to report on opioid use and cost associated with total knee arthroplasty (TKA) following tibial plateau fracture fixation as compared to primary TKA patients with no history of tibial plateau fracture. The aim of this study is to compare the differences in opioid use, reimbursements, and complication rates between patients with and without a history of tibial plateau fracture undergoing TKA.

Methods and materials

This is a* *retrospective large database review study. The study included patients across the country and in various clinical settings including, but not limited to, institutions, primary and tertiary care centers, and private practice. The PearlDiver database was reviewed for patients undergoing TKA between 2010 and 2019. Patients who underwent TKA following surgical repair of a tibial plateau fracture were identified using Common Procedural Terminology (CPT) codes and the appropriate International Classification of Diseases Ninth and Tenth Revision (ICD-9, ICD-10) codes. This group was then matched by age, gender, Charleston Comorbidity Index (CCI) score, Elixhauser Comorbidity Index (ECI) score, obesity, tobacco use, and diabetes to a group of similar patients who underwent TKA with no history of tibial plateau fracture. Opioid use over the episode of care, evaluated by morphine milligram equivalents (MME), and 30-day reimbursed cost were compared between groups using an unequal variance t-test. Complication rates at 30 days, 90 days, and one year postoperatively, and revision rates at one and two years postoperatively were compared using the odd’s ratio (OR) with 95% confidence intervals (95%CI).

Results

The episode of care cost for TKA was significantly lower for patients with a history of tibial plateau fracture ($11,615 ± $15,704) than it was for patients without a history of tibial plateau fracture ($16,088 ± $18,573) (p = 3.56E-14). At 30 days after knee arthroplasty, patients with a history of tibial plateau fracture had significantly more episodes of dehiscence (OR 2.665 [95% CI 1.327-5.351]; p = 0.006) and surgical site infection (SSI) (OR 1.698 [95% CI 1.058-2.724]; p = 0.028), which was significant at 90 days postop for both dehiscence (OR 1.358 [95% CI 0.723-2.551]; p = 0.001) and SSI (OR 1.634 [95% CI 1.100-1.802]; p = 0.015), as well as mechanical complications of the implant device (OR 2.420 [95% CI 1.154-5.076]; p = 0.019). There was no significant difference in the number of opioids prescribed postoperatively to patients with a history of tibial plateau fracture (2218 ± 3255 MME) compared to those without prior tibial plateau fracture (2400 ± 4843 MME) (p = 0.258). However, there was a small but statistically significant increase in the number of days postoperatively patients with a history of tibial plateau fracture were prescribed opioids (11.99 ± 7.73 days) compared to non-tibial plateau fracture patients (11.15 ± 7.18 days) (p = 0.004).

Conclusion

Patients with a history of tibial plateau fracture who then underwent conversion TKA have a lower reimbursed cost of TKA but a higher postoperative risk for dehiscence, mechanical complications, and surgical site infections. There is no significant difference in postoperative opioid use between the two groups.

## Introduction

Many studies have shown that patients who have had surgical repair of a tibial plateau fracture are at increased risk of conversion to total knee arthroplasty (TKA) [1-9]. Often, these patients present with stiffness, pre-existing surgical incisions, orthopedic implants in place, cartilage damage, and possible malunion with or without bony defects [2,10]. This previous tissue damage, along with any implanted hardware from tibial plateau fracture fixation, increases the complexity of conversion to TKA and thus also increases complication rates [7,11-12]. The increased complexity of conversion TKA has also been shown to increase operative time [11]. The combination of increased operative time and perioperative complications likely increases costs for orthopedic surgeons and surgical centers performing these procedures. Despite the increased difficulty of conversion TKA, Current Procedural Terminology (CPT) coding currently does not distinguish between TKA and conversion TKA, which may create an adverse incentive for orthopedic surgeons to perform complex conversion TKA surgeries [11]. There is a paucity of data on reimbursement for primary TKA compared to conversion TKA. Data on opioid requirements following conversion TKA compared to primary TKA are also limited. Further, despite the existence of several institutional studies on conversion TKA complication rates, few, if any, large database studies exist to compare complications across a large number of patients undergoing conversion TKA versus a matched cohort of patients undergoing primary TKA.

Our purpose is to determine the difference in reimbursed cost, opioid use, and complication rates following total knee arthroplasty with or without a history of tibial plateau fracture fixation. We hypothesized that conversion TKA would be associated with higher postoperative complication rates, opioid use, and reimbursement.

## Materials and methods

In this Level III retrospective cohort analysis, we used the PearlDiver all-payer claims database (www.pearldiverinc.com, Colorado Springs, CO, USA). PearlDiver is a commercially available, nationwide database with medical and prescription records for over 137 million patients from all payer types, including commercial insurance, Medicare, Medicaid, and self-pay. All information is de-identified and is compliant with the Health Insurance Portability and Affordability Act (HIPAA). Because the de-identified data are publicly available, this study did not require approval by an institutional review board (IRB).

The PearlDiver database was reviewed for patients undergoing TKA between the years 2010 and 2019. Patients were identified using appropriate International Classification of Disease Ninth and Tenth Revision (ICD-9, ICD-10) codes and Current Procedural Terminology (CPT) codes (Appendix). Patients were then stratified into two cohorts. The first cohort were those who had a record indicating surgical fixation of a tibial plateau fracture within two years prior to undergoing TKA, identified by CPT codes. This group was then matched to a similar cohort of TKA patients with no history of tibial plateau fracture repair. Matching was done by the PearlDiver Bellweather program, using the Charlson Comorbidity Index (CCI), Elixhauser Comorbidity Index (ECI), age, gender, obesity, tobacco smoking history, and diabetes status as matching parameters. Complications, identified using appropriate ICD codes, were also collected and analyzed at 30 days, 90 days, and one year postoperatively. To ensure complication rates were not biased by patient dropouts from the database (i.e. patients switching insurance companies during the follow-up period), all patients who were not active for at least two years prior to TKA and who did not remain active in the database for at least one year following TKA were excluded from the analysis. Revision of TKA was also analyzed at two years postoperatively using only patients who remained active in the database for at least two years following TKA to avoid bias as described above.

As the PearlDiver program reports -1 for all number of complications fewer than 11 within a group, when either the tibial plateau or non-tibial plateau group had 11 or more complications, with 10 or fewer in the corresponding matched group, the 10 group was assigned a value of 10, in order to allow for analysis while also minimizing the risk of Type 1 error. Sixty-day opioid use following TKA, as identified using appropriate Uniform System of Classification (USC) drug codes (Appendix), was evaluated by morphine milligram equivalent (MME). The reimbursed episode of care cost was recorded and evaluated as well.

Statistics

The R statistical program (Core Team (2020). R: A language and environment for statistical computing. R Foundation for Statistical Computing, Vienna, Austria) provided through PearlDiver was used for statistical analysis of our data. Opioid use and cost of care were compared between groups using the Welch unequal variance t-test. Complication rates at 30 days, 90 days, and one year postoperatively were compared using odd’s ratio (OR) with 95% confidence intervals (95%CI).

## Results

A total of 954,021 patients underwent TKA between 2010 and 2019 and remained active in the database for at least two years prior to and one year following TKA. During the same time period, 3,929 patients underwent surgical repair of a tibial plateau fracture. Of those patients, 1,750 (45%) underwent conversion to TKA within two years. We were able to match 1,712 of these tibial plateau conversions to TKA patients with 1,712 primary TKA patients without a history of tibial plateau fracture using CCI, ECI, smoking history, diabetes status, age, gender, and obesity as match parameters. Thus, our study includes a total of 3,424 patients (1,712 in each matched group, 29% male and 71% female). As the patients were matched using these parameters, there were no significant differences between the groups by any demographic or comorbidity variable (Table 1). Complete patient-level data required to evaluate postoperative opioid use was available for 1,299 patients in the conversion TKA and 1,326 patients in the primary TKA group.

**Table 1:** Summary demographic data of the total and sample patient populations of the study CCI: Charlson Comorbidity Index; ECI: Elixhauser Comorbidity Index

		All TKA patients	Matched cohort with prior tibial plateau fracture	Matched cohort without prior tibial plateau fracture
Total Patients		954,021	1712	1712
Male		349,763 (36.7%)	503 (29.4%)	503 (29.4%)
Female		604,258 (63.3%)	1209 (70.6%)	1209 (70.6%)
Average Age		65.9 (± 8.8)	63.5 (± 9.5)	63.6 (± 9.4)
Average CCI score		0.64 (±1.31)	1.17 (± 1.76)	1.16 (± 1.76)
Average ECI score		4.38 (±3.55)	7.1 (± 3.9)	7.1 (± 3.9)
Obese	Yes	484,641 (50.8%)	821 (48.0%)	821 (48.0%)
	No	469,380 (49.2%)	891 (52.0%)	891 (52.0%)
Diabetes	Yes	450,896 (47.3%)	778 (45.4%)	778 (45.4%)
	No	503,125 (52.7%)	934 (54.6%)	934 (54.6%)
Smoking	Yes	230,704 (24.2%)	555 (32.4%)	555 (32.4%)
	No	723,317 (75.8%)	1157 (67.6%)	1157 (67.6%)

Complications

Patients with conversion to TKA following surgical repair of a tibial plateau fracture have a significantly increased rates of wound dehiscence at 30 days (OR 2.665 [95% CI 1.327-5.351]; p = 0.0059), 90 days (OR 1.358 [95% CI 0.723-2.551]; p = 0.001), and one year (OR 2.073 [95% CI 1.253-3.429]; p = .005) after TKA, increased rates of surgical site infection (SSI) at 30 days (OR 1.698 [95% CI 1.058-2.724]; p = 0.028), 90 days (OR 1.634 [95% CI 1.100-1.802]; p = 0.015), and one year (OR 1.813 [95% CI 1.282-2.565] p = .0008) after TKA, and increased rates of mechanical complications of implanted devices at 90 days (OR 2.420 [95% CI 1.154-5.076]; p = 0.019) and one year (OR 1.817 [95% CI 1.048-3.152] p = 0.034) after TKA. There was no significant difference found in revision rates at one (OR 1.204 [95%CI 0.738-1.964] p = 0.456) or two (OR 1.496 [95%CI 0.983-2.277] p = 0.060) years following TKA, nor were there increased rates at one year of stiffness, manipulation under anesthesia (MUA), component loosening, or periprosthetic fracture (Table 2).

**Table 2:** 30-day, 90-day, and one-year complication rate after knee arthroplasty stratified by tibial plateau fracture history. Odds ratio, 95% confidence interval, and p-values presented. * = statistically significant at p<0.05 level. **Incidence ≤10 but set at 10 to allow for statistical analysis. N/A = Not available (If one of the cells is 0 or if the output is not given since it is less than 10, it is not possible to calculate the odds ratio and P-value)

Complication		Prior Tibial Plateau Fracture	No Tibial Plateau Fracture	Odds Ratio	Lower 95% CI	Upper 95% CI	P-value
10-day Presumed Mortality		≤10	0	N/A	N/A	N/A	N/A
Acute Kidney Injury	30 day	28	20	1.407	0.789	2.507	0.247
	90 day	43	32	1.353	0.852	2.148	0.201
	1 year	84	64	1.329	0.953	1.852	0.094
Cardiac Arrest	30 day	≤10	≤10	N/A	N/A	N/A	N/A
	90 day	≤10	≤10	N/A	N/A	N/A	N/A
	1 year	≤10	≤10	N/A	N/A	N/A	N/A
Cerebrovascular Accident	30 day	≤10	≤10	N/A	N/A	N/A	N/A
	90 day	≤10	≤10	N/A	N/A	N/A	N/A
	1 year	10**	11	0.909	0.385	2.145	0.827
Deep Vein Thrombosis	30 day	≤10	≤10	N/A	N/A	N/A	N/A
	90 day	11	10**	1.101	0.466	2.598	0.827
	1 year	23	17	1.358	0.723	2.551	0.342
Dehiscence	30 day	29	11	2.665	1.327	5.351	0.006*
	90 day	42	17	2.508	1.422	4.423	0.002*
	1 year	47	23	2.073	1.253	3.429	0.005*
Hematoma	30 day	13	11	1.183	0.529	2.649	0.682
	90 day	17	15	1.135	0.565	2.279	0.723
	1 year	21	21	1.000	0.544	1.838	1.000
Myocardial Infarction	30 day	13	12	1.084	0.493	2.382	0.841
	90 day	20	18	1.112	0.586	2.110	0.744
	1 year	45	43	1.048	0.686	1.600	0.829
Nerve Injury	30 day	0	0	N/A	N/A	N/A	N/A
	90 day	0	0	N/A	N/A	N/A	N/A
	1 year	≤10	0	N/A	N/A	N/A	N/A
Pneumonia	30 day	21	18	1.169	0.620	2.201	0.629
	90 day	28	23	1.221	0.700	2.128	0.481
	1 year	69	53	1.315	0.913	1.893	0.141
Pulmonary Embolism	30 day	14	13	1.078	0.505	2.299	0.847
	90 day	21	17	1.238	0.651	2.355	0.515
	1 year	29	26	1.117	0.655	1.905	0.684
Sepsis	30 day	14	10**	1.403	0.622	3.168	0.415
	90 day	17	14	1.216	0.598	2.476	0.589
	1 year	29	27	1.075	0.634	1.824	0.788
Surgical Site Infection	30 day	47	28	1.698	1.058	2.724	0.028*
	90 day	66	41	1.634	1.100	2.427	0.015*
	1 year	92	52	1.813	1.282	2.565	0.001*
Transfusion	30 day	44	28	1.587	0.983	2.560	0.059
	90 day	47	32	1.482	0.941	2.334	0.090
	1 year	54	45	1.207	0.808	1.802	0.359
Stiffness	1 year	70	93	0.742	0.540	1.020	0.066
Stiffness then Manipulation Under Anesthesia	1 year	17	29	0.582	0.319	1.063	0.078
Total Manipulation Under Anesthesia	1 year	84	88	0.952	0.701	1.294	0.754
Periprosthetic Fracture	1 year	≤10	≤10	N/A	N/A	N/A	N/A
Component Loosening	1 year	≤10	≤10	N/A	N/A	N/A	N/A
Revision	1 year	36	30	1.204	0.738	1.964	0.456
	2 years	53	39	1.496	0.983	2.277	0.060

Episode-of-care cost

Patients undergoing conversion TKA after surgical repair of a tibial plateau fracture had a statistically significantly lower reimbursed cost of care associated with TKA than patients undergoing primary TKA ($11,615 ± $15,704 for conversion TKA versus $16,088 ± $18573 for primary TKA, p < 0.0001). There was, however, no difference in length of stay between the two groups (2.80 ± 1.49 days for conversion TKA versus 2.88 ± 1.55 days for primary TKA, p = 0.217).

Postoperative opioid use

There was no difference in the MME of opioids prescribed to conversion TKA patients compared to the matched primary TKA cohort (2218 ± 3255 MME for conversion TKA versus 2400 ± 4843 MME for primary TKA, p = 0.258). Although the total MME of opioids was not significantly different between the groups, patients who had a history of tibial plateau fracture receiving opioids for approximately one more day than those with no history of tibial plateau fracture (11.98 ± 7.73 days for conversion TKA patients versus 11.15 ± 7.18 days for primary TKA patients, p = 0.004). 

## Discussion

The main findings of this study are that patients undergoing conversion TKA following prior surgical repair of a tibial plateau fracture are at an increased risk of surgical site complications at 30 days and both surgical site and mechanical complications of implanted devices at 90 days and in the first postoperative year. It is likely that the difference in rates of mechanical complications was significant at 30 days and perhaps even more significant than indicated at 90 days, but because there were 10 mechanical complications in the primary TKA group at each of these periods, we could not accurately compare the exact ratios. As explained previously, we substituted 10 as the number of mechanical complications for the primary TKA group at these intervals, which yielded the reported results, but the actual results may have been more significant. However, despite being more complicated and prone to higher complication rates, conversion TKA after tibial plateau fracture repair was reimbursed less than primary TKA. in this matched cohort. There was no difference in MME opioid prescription between the two groups.

Open reduction with internal fixation (ORIF) is currently the standard of care for tibial plateau fractures [6]. However, patients who undergo this procedure are at an increased risk of postoperative complications. The most common complication following a tibial plateau fracture is post-traumatic knee osteoarthritis (PTOA), which has been reported in 13%-83% of patients [2,5-6,13-15]. Deep infection was also reported in 14.2% of patients who underwent ORIF for a tibial plateau fracture [16]. Scott et al. found that varus or valgus collapse with resultant malunion was another common complication of tibial plateau fractures [17]. Several studies have shown that these complications lead to an increased risk of conversion to TKA following ORIF for tibial plateau fracture [2,5-6,13-17]. 

While 45% of patients undergoing surgical repair of a tibial plateau fracture progressed to conversion TKA in our study, caution must be used in interpreting this ratio, as it is significantly higher than the rate of conversion found in other studies. Our data were obtained from a database that includes primarily records from patients who have undergone knee arthroplasty. Thus, we are unable to comment on the rate of conversion to TKA following ORIF for tibial plateau fracture in the general population. This was intentional, as our study is primarily focused on the complications and outcomes of secondary TKA following the failure of tibial plateau fracture repair, and we aimed to maximize the number of patients with conversion to TKA.

As mentioned previously, other studies examining the rate of conversion to TKA following a tibial plateau fracture have found significantly lower conversion rates, though rates of conversion TKA in patients with tibial plateau fracture are still significantly higher than the rate of TKA in the general population [2,5,9]. Wasserstein et al. found that conversion TKA occurred in 7.3% of tibial plateau fracture patients within 10 years, which is up to five times higher than matched controls from the general population with a conversion rate of 1.8% [9]. Elsoe et al. found a slightly lower conversion rate of 5.7% in tibial plateau patients compared to 2% of the general population of similar age and gender who undergo primary TKA [2]. Scott et al. found conversion to TKA amongst tibial plateau fracture patients to be 4.7% within five years. However, other researchers have found that conversion TKA is a relatively uncommon procedure following ORIF of tibial plateau fracture [18-19]. Pinter et al. found that conversion to TKA following a tibial plateau fracture occurs in only 2.15% of patients [18].

Often, these patients present with stiffness, pre-existing surgical incisions, orthopedic implants in place, cartilage damage, and possible malunion with or without bony defects [2,10]. This previous tissue damage, along with any implanted hardware from tibial plateau fracture repair increases the complexity of conversion to TKA [7]. Alterations to the soft tissue environment from previous surgeries have been shown to complicate wound healing in future procedures at the same location. Our study confirms this, as 47 (2.7%) patients with conversion TKA had complications of dehiscence within one year, which was significantly more than the 23 (1.3%) of primary TKA patients with dehiscence within one year (p = 0.005). Patients in our study undergoing conversion TKA also had significantly higher rates of surgical site infection within one year (92 patients, 5.4%) than patients undergoing primary TKA (52 patients, 3.0%) (p = .001). Previous studies have also reported higher rates of infection among conversion TKA patients, with surgical site infection reported in 3% to 26% of conversion TKA patients [8,18,20-25].

We also found mechanical complications involving orthopedic implant devices or grafts were more common in conversion TKA patients than in primary TKA patients. The increased complication rate was significant at 90 days (OR 2.42 [95% CI 1.154-5.076] p = 0.019) and one year (OR 1.817 [95% CI 1.048-3.152] p = 0.034). There were 10 patients with mechanical complications in the primary TKA group at both 30 and 90 days (compared to 16 and 24 patients in the conversion TKA group at 30 and 90 days, respectively), so it is plausible that the difference in mechanical complication rates was significant at 30 days but due to the limitations of this database study, we cannot confirm this.

Despite increased complication rates found in conversion TKA patients, we found no significant difference in the revision of TKA after one year. Although the difference in the rate of revision at two years did not reach statistical significance (OR 1.496 [95%CI 0.983-2.277] p = 0.060), we feel this difference is clinically meaningful. Patients who underwent conversion TKA were nearly 1.5 times more likely to require revision TKA. We were unable to tell if there was a difference in rates of periprosthetic fracture or component loosening within one year, as these events were extremely rare, both occurring in 10 patients in each group. These results are also consistent with several previous studies, which have found excellent to good overall and functional outcomes in the majority of secondary TKA patients despite increased complications [17,22,24,26]. However, they are inconsistent with Pinter et al.’s single-institution study, which found that four out of 20 (20%) of patients with conversion TKA required revision surgery [18].

Interestingly, there is a strong trend indicating that conversion TKA was protective against MUA and stiffness, although not significantly so. We speculate this is likely because conversion TKA patients were already experiencing stiffness preoperatively and hence stiffness after surgery was better tolerated by these patients, thereby leading to less MUA.

The combination of increased operative time and complications for conversion TKA increases costs for surgical centers and hospitals performing these procedures. Despite this increased complexity, CPT coding currently does not distinguish between TKA and conversion TKA, which may create an adverse incentive for orthopedic surgeons to perform conversion TKA surgeries [11]. There is a paucity of data in the literature on reimbursement for primary TKA compared to conversion TKA. We found that reimbursement for conversion TKA is significantly lower than reimbursement for primary TKA ($11,615 ± $15,704 for conversion TKA versus $16,088 ± $18,573 for primary TKA, p = 3.56E-14). Unfortunately, we cannot determine how much either operation cost in materials, OR time, or physician time for comparison, but Bergen et al. have shown that conversion TKA takes longer than primary TKA and has higher operative and post-acute resource utilization [11]. Due to the limitations of database studies, we cannot determine why reimbursement was lower for this group. However, our study is unique in addressing this point and identifies an area where further research is warranted to determine if there truly is a disincentive for orthopedic surgeons to practice and take on complex conversion TKA procedures.

This study is also unique in looking at opioid prescriptions by MME between these two cohorts. Interestingly, there was no significant difference in the total MME of opioids prescribed to both groups within 60 days of TKA (2218 ± 3255 MME for conversion TKA versus 2400 ± 4843 MME for primary TKA, p = 0.258). Although the total MME of opioids was not different between the groups, patients who underwent conversion TKA received opioids for approximately one more day than those who underwent primary TKA (11.98 ± 7.73 days for conversion TKA patients versus 11.15 ± 7.18 days for primary TKA patients, p = 0.004), though this short time difference may not be clinically significant. An important observation of this study is that despite conversion TKA patients having more complications, they were not prescribed more opioids to manage any associated pain. However, the database does not provide data to determine how much of these opioids were actually consumed by patients, nor can we state whether or not patients’ pain was adequately managed with these narcotic regimens. Further, the difference appears to be clinically insignificant. This is perhaps another area where further research may be warranted.

This study has an advantage over previous studies in that all of the patients were matched to similar patients based on age, gender, smoking history, obesity, diabetes, CCI, and ECI. This is important, as smoking is associated with an increased risk for postoperative infection [27] and infection with ORIF is associated with additional surgeries [6,16]. Wasserstein et al. have also shown that comorbidities and age also strongly correlate with an increased risk of early-onset PTOA [9], which increases the risk of conversion to TKA [2]. Scott et al. also found that female gender and obesity were significant risk factors for conversion to TKA [5]. Previous studies have matched various combinations of these comorbidities, but none have had the patient volume to match a comprehensive list of risk factors and comorbidities.

Limitations

Our study is not without limitations. While PearlDiver allows for the aggregation of large amounts of data, we are limited to what is coded through this database. The PearlDiver database’s reliance on accurate ICD and CPT codes, as well as billing information, creates the potential for reporting bias. Further, there is a lack of coding laterality in database studies that may have caused the overestimation of some values by including patients in the conversion TKA group who actually had a primary TKA in the contralateral knee to their tibial plateau fracture. This could potentially have lowered the complication rates of conversion TKA, possibly masking significant findings in regards to complications or revisions. Additionally, there is a lack of granularity inherent to large database studies that precludes our ability to stratify results based on types of implant used, type of tibial plateau repair procedure, or one versus two-stage conversion TKA. Another limitation is that it does report on hospital or surgeon volume, potentially an important variable in assessing complication rates. Finally, there is a possibility that this study is subject to Type II error, as it may be underpowered to detect a significant difference in the revision rates within two years (p = 0.060), stiffness within one year (p = 0.066), or transfusion within 30 days (p = 0.059), each of which has been shown to be significant complications in previous studies [2,5,9,11,26,28-29].

## Conclusions

In conclusion, we found that patients undergoing conversion TKA after a previously repaired tibial plateau fracture were more likely to suffer complications, including dehiscence and surgical site infection at 30 days, 90 days, and one year postoperatively, and had an increased rate of mechanical complications at 90 days and one year postoperatively. The reimbursed cost of TKA was lower in conversion TKA patients following tibial plateau fracture repair than in a matched cohort of primary TKA patients. There was no difference in length of stay between the two groups. Further, there was no significant difference in the MME of opioid use between the two matched groups, though patients with a history of tibial plateau fractures were prescribed lower daily doses for approximately one day longer on average.

## Appendices

Appendix A: International Classification of Diseases, Ninth and Tenth Revision, Clinical Modification (ICD-9-CM, ICD-10-CM), Current Procedural Terminology codes & Uniform System of Classification for pharmacological products

ICD-9-CM Diagnosis Code, ICD-10-CM Diagnosis Code, CPT Code, USC Drug Codes

Acute Kidney Injury (AKI)

ICD-9-D-5845,ICD-9-D-5846,ICD-9-D-5847,ICD-9-D-5848,ICD-9-D-5849,ICD-10-D-N17:ICD-10-D-N179

Cannabis Use, Abuse, or Dependence

ICD-10-D-F12120, ICD-10-D-F12121, ICD-10-D-F12122, ICD-10-D-F12129, ICD-10-D-F12150, ICD-10-D-F12151, ICD-10-D-F12159, ICD-10-D-F12180, ICD-10-D-F12188, ICD-10-D-F1219, ICD-10-D-F1220, ICD-10-D-F12220, ICD-10-D-F12222, ICD-10-D-F12229, ICD-10-D-F1223, ICD-10-D-F12250, ICD-10-D-F12251, ICD-10-D-F12259, ICD-10-D-F12280, ICD-10-D-F12288, ICD-10-D-F1229, ICD-10-D-F1290, ICD-10-D-F12920, ICD-10-D-F12921, ICD-10-D-F12922, ICD-10-D-F12929, ICD-10-D-F1293, ICD-10-D-F12950, ICD-10-D-F12951, ICD-10-D-F12959, ICD-10-D-F12980, ICD-10-D-F12988, ICD-10-D-F1299, ICD-10-D-F1210, ICD-9-D-30430, ICD-9-D-30431, ICD-9-D-30432, ICD-9-D-30520, ICD-9-D-30521, ICD-9-D-30522

Cardiac Arrest

ICD-9-D-4275,ICD-9-D-42741,ICD-10-D-I46:ICD-10-D-I469

Cerebrovascular Accident (CVA)

ICD-10-D-I97821, ICD-9-D-43491

Component Loosening, Knee

ICD-10-D-T84032A, ICD-10-D-T84033A

Deep Vein Thrombosis

ICD-9-D-45340, ICD-10-D-I82409, ICD-9-D-45341, ICD-10-D-I82419, ICD-10-D-I82429, ICD-10-D-I82439, ICD-10-D-I824Y9, ICD-9-D-45341, ICD-10-D-I82419, ICD-10-D-I82429, ICD-10-D-I82439, ICD-10-D-I824Y9, ICD-9-D-45381, ICD-10-D-I82619, ICD-9-D-45382, ICD-10-D-I82629, ICD-9-D-45383, ICD-10-D-I82609, ICD-9-D-45384, ICD-10-D-I82A19, ICD-9-D-45385, ICD-10-D-I82B19, ICD-9-D-45386, ICD-10-D-I82C19, ICD-9-D-45387, ICD-10-D-I82290, ICD-9-D-45389, ICD-10-D-I82890, ICD-9-D-4539, ICD-10-D-I8291, ICD-9-D-41512, ICD-10-D-I2690, ICD-9-D-41513, ICD-10-D-I2692, ICD-9-D-41519, ICD-10-D-I2699

Dehiscence

ICD-9-D-99830,ICD-9-D-99831,ICD-9-D-99832,ICD-9-D-99833,ICD-10-D-T8130XA,ICD-10-D-T8130XD,ICD-10-D-T8130XS,ICD-10-D-T8131XA,ICD-10-D-T8131XD,ICD-10-D-T8131XS,ICD-10-D-T8132XA,ICD-10-D-T8132XD,ICD-10-D-T8132XS,ICD-10-D-T8133XA,ICD-10-D-T8133XD,ICD-10-D-T8133XS

Hematoma

ICD-9-D-99811,ICD-9-D-99812,ICD-9-D-99813,ICD-10-D-D7801, ICD-10-D-D7802, ICD-10-D-D7821, ICD-10-D-D7822, ICD-10-D-E3601, ICD-10-D-E3602, ICD-10-D-E89810, ICD-10-D-E89811, ICD-10-D-G9731, ICD-10-D-G9732, ICD-10-D-G9751, ICD-10-D-G9752, ICD-10-D-H59111, ICD-10-D-H59112, ICD-10-D-H59113, ICD-10-D-H59119, ICD-10-D-H59121, ICD-10-D-H59122, ICD-10-D-H59123, ICD-10-D-H59129, ICD-10-D-H59311, ICD-10-D-H59312, ICD-10-D-H59313, ICD-10-D-H59319, ICD-10-D-H59321, ICD-10-D-H59322, ICD-10-D-H59323, ICD-10-D-H59329, ICD-10-D-H9521, ICD-10-D-H9522, ICD-10-D-H9541, ICD-10-D-H9542, ICD-10-D-I97410, ICD-10-D-I97411, ICD-10-D-I97418, ICD-10-D-I9742, ICD-10-D-I97610, ICD-10-D-I97611, ICD-10-D-I97618, ICD-10-D-I97620, ICD-10-D-J9561, ICD-10-D-J9562, ICD-10-D-J95830, ICD-10-D-J95831, ICD-10-D-K9161, ICD-10-D-K9162, ICD-10-D-K91840, ICD-10-D-K91841, ICD-10-D-L7601, ICD-10-D-L7602, ICD-10-D-L7621, ICD-10-D-L7622, ICD-10-D-M96810, ICD-10-D-M96811, ICD-10-D-M96830, ICD-10-D-M96831, ICD-10-D-N9961, ICD-10-D-N9962, ICD-10-D-N99820, ICD-10-D-N99821, ICD-10-D-T888XXA

Knee Stiffness

ICD-9-D-71956

Manipulation Under Anesthesia

CPT-27570

Mechanical Complication

ICD-9-D-99659, ICD-9-D-99649, ICD-9-D-99647, ICD-9-D-99640

Myocardial Infarction

ICD-9-D-41000, ICD-9-D-41001, ICD-9-D-41002, ICD-9-D-41010, ICD-9-D-41011, ICD-9-D-41012, ICD-9-D-41020, ICD-9-D-41021, ICD-9-D-41022, ICD-9-D-41030, ICD-9-D-41031, ICD-9-D-41032, ICD-9-D-41040, ICD-9-D-41041, ICD-9-D-41042, ICD-9-D-41050, ICD-9-D-41051, ICD-9-D-41052, ICD-9-D-41080, ICD-9-D-41081, ICD-9-D-41082, ICD-9-D-41090, ICD-9-D-41091, ICD-9-D-41092, ICD-10-D-I2101, ICD-10-D-I2102, ICD-10-D-I2109, ICD-10-D-I2111, ICD-10-D-I2119, ICD-10-D-I2121, ICD-10-D-I2129, ICD-10-D-I213, ICD-10-D-I214, ICD-10-D-I219, ICD-10-D-I21A1, ICD-10-D-I21A9, ICD-10-D-I220, ICD-10-D-I221, ICD-10-D-I222, ICD-10-D-I228, ICD-10-D-I229

Nerve Injury

ICD-9-D-9550,ICD-9-D-9551,ICD-9-D-9552,ICD-9-D-9553,ICD-9-D-9554,ICD-9-D-9555,ICD-9-D-9556,ICD-9-D-9557,ICD-9-D-9558,ICD-9-D-9559,ICD-9-D-9074,ICD-10-D-S440,ICD-10-D-S4400,ICD-10-D-S4400XA,ICD-10-D-S4400XD,ICD-10-D-S4400XS,ICD-10-D-S4401,ICD-10-D-S4401XA,ICD-10-D-S4401XD,ICD-10-D-S4401XS,ICD-10-D-S4402,ICD-10-D-S4402XA,ICD-10-D-S4402XD,ICD-10-D-S4402XS,ICD-10-D-S441,ICD-10-D-S4410,ICD-10-D-S4410XA,ICD-10-D-S4410XD,ICD-10-D-S4410XS,ICD-10-D-S4411,ICD-10-D-S4411XA,ICD-10-D-S4411XD,ICD-10-D-S4411XS,ICD-10-D-S4412,ICD-10-D-S4412XA,ICD-10-D-S4412XD,ICD-10-D-S4412XS,ICD-10-D-S442,ICD-10-D-S4420,ICD-10-D-S4420XA,ICD-10-D-S4420XD,ICD-10-D-S4420XS,ICD-10-D-S4421,ICD-10-D-S4421XA,ICD-10-D-S4421XD,ICD-10-D-S4421XS,ICD-10-D-S4422,ICD-10-D-S4422XA,ICD-10-D-S4422XD,ICD-10-D-S4422XS,ICD-10-D-S443,ICD-10-D-S4430,ICD-10-D-S4430XA,ICD-10-D-S4430XD,ICD-10-D-S4430XS,ICD-10-D-S4431,ICD-10-D-S4431XA,ICD-10-D-S4431XD,ICD-10-D-S4431XS,ICD-10-D-S4432,ICD-10-D-S4432XA,ICD-10-D-S4432XD,ICD-10-D-S4432XS,ICD-10-D-S444,ICD-10-D-S4440,ICD-10-D-S4440XA,ICD-10-D-S4440XD,ICD-10-D-S4440XS,ICD-10-D-S4441,ICD-10-D-S4441XA,ICD-10-D-S4441XD,ICD-10-D-S4441XS,ICD-10-D-S4442,ICD-10-D-S4442XA,ICD-10-D-S4442XD,ICD-10-D-S4442XS,ICD-10-D-S445,ICD-10-D-S4450,ICD-10-D-S4450XA,ICD-10-D-S4450XD,ICD-10-D-S4450XS,ICD-10-D-S4451,ICD-10-D-S4451XA,ICD-10-D-S4451XD,ICD-10-D-S4451XS,ICD-10-D-S4452,ICD-10-D-S4452XA,ICD-10-D-S4452XD,ICD-10-D-S4452XS,ICD-10-D-S448,ICD-10-D-S448X,ICD-10-D-S448X1,ICD-10-D-S448X1A,ICD-10-D-S448X1D,ICD-10-D-S448X1S,ICD-10-D-S448X2,ICD-10-D-S448X2A,ICD-10-D-S448X2D,ICD-10-D-S448X2S,ICD-10-D-S448X9,ICD-10-D-S448X9A,ICD-10-D-S448X9D,ICD-10-D-S448X9S,ICD-10-D-S449,ICD-10-D-S4490,ICD-10-D-S4490XA,ICD-10-D-S4490XD,ICD-10-D-S4490XS,ICD-10-D-S4491,ICD-10-D-S4491XA,ICD-10-D-S4491XD,ICD-10-D-S4491XS,ICD-10-D-S4492,ICD-10-D-S4492XA,ICD-10-D-S4492XD,ICD-10-D-S4492XS

Opioid Use

USC-02211, USC-02212, USC-02214, USC-02221, USC-02222, USC-02232

Periprosthetic Fracture, Knee

ICD-10-D-M9711XA, ICD-10-D-M9712XA, ICD-10-D-T84042A, ICD-10-D-T84043A

Pneumonia

ICD-9-D-4800:ICD-9-D-4809,ICD-9-D-481,ICD-9-D-4820,ICD-9-D-4821,ICD-9-D-48230,ICD-9-D-48231,ICD-9-D-48232,ICD-9-D-48239,ICD-9-D-48240,ICD-9-D-48241,ICD-9-D-48242,ICD-9-D-48249,ICD-9-D-48281,ICD-9-D-48282,ICD-9-D-48283,ICD-9-D-48284,ICD-9-D-48289,ICD-9-D-4829,ICD-9-D-4830,ICD-9-D-4831,ICD-9-D-4838,ICD-9-D-4841,ICD-9-D-4843,ICD-9-D-4845,ICD-9-D-4846,ICD-9-D-4847,ICD-9-D-4848,ICD-9-D-485,ICD-9-D-486,ICD-10-D-J12:ICD-10-D-J189

Pulmonary Embolism

ICD-9-D-4151:ICD-9-D-4159,ICD-10-D-I26:ICD-10-D-I269

Revision of TKA

ICD-9-P-0084, ICD-9-P-0080, ICD-9-P-0081, ICD-9-P-0083, ICD-9-P-8155, ICD-9-P-0082, CPT-27486, CPT-27487

Sepsis

ICD-9-D-99591, ICD-10-D-A419

Surgical Site Infection

ICD-10-D-T814,ICD-10-D-T814XXA,ICD-10-D-T814XXD,ICD-10-D-M86:ICD-10-D-M869,ICD-10-D-M868X0,ICD-10-D-M868X1,ICD-10-D-M868X2,ICD-10-D-M868X3,ICD-10-D-M868X4,ICD-10-D-M868X5,ICD-10-D-M868X6,ICD-10-D-M868X7,ICD-10-D-M868X8,ICD-10-D-M868X9,ICD-10-D-T814XXS,ICD-9-D-99851,ICD-9-D-99859,ICD-9-D-99666,ICD-9-D-99667,ICD-9-D-73001,ICD-9-D-73011,ICD-9-D-73021,ICD-9-D-73081,ICD-9-D-73091

Tibial Plateau Fracture Repair

CPT-27530, CPT-27532, CPT-27535, CPT-27536, CPT-29855, CPT-29856

Total Knee Arthroscopy

ICD-9-P-8154, ICD-10-P-0SRC069, ICD-10-P-0SRC06A, ICD-10-P-0SRC06Z, ICD-10-P-0SRC0J9, ICD-10-P-0SRC0JA, ICD-10-P-0SRC0JZ, ICD-10-P-0SRD069, ICD-10-P-0SRD06A, ICD-10-P-0SRD06Z, ICD-10-P-0SRD0J9, ICD-10-P-0SRD0JA, ICD-10-P-0SRD0JZ, ICD-10-P-0SRT0J9, ICD-10-P-0SRT0JA, ICD-10-P-0SRT0JZ, ICD-10-P-0SRU0J9, ICD-10-P-0SRU0JA, ICD-10-P-0SRU0JZ, ICD-10-P-0SRV0J9, ICD-10-P-0SRV0JA, ICD-10-P-0SRV0JZ, ICD-10-P-0SRW0J9, ICD-10-P-0SRW0JA, ICD-10-P-0SRW0JZ, CPT-27447

Transfusion

ICD-9-P-9904,ICD-10-P-3023,ICD-10-P-30230AZ,ICD-10-P-30230G0,ICD-10-P-30230G2,ICD-10-P-30230G3,ICD-10-P-30230G4,ICD-10-P-30230H0,ICD-10-P-30230H1,ICD-10-P-30230J0,ICD-10-P-30230J1,ICD-10-P-30230K0,ICD-10-P-30230K1,ICD-10-P-30230L0,ICD-10-P-30230L1,ICD-10-P-30230M0,ICD-10-P-30230M1,ICD-10-P-30230N0,ICD-10-P-30230N1,ICD-10-P-30230P0,ICD-10-P-30230P1,ICD-10-P-30230Q0,ICD-10-P-30230Q1,ICD-10-P-30230R0,ICD-10-P-30230R1,ICD-10-P-30230S0,ICD-10-P-30230S1,ICD-10-P-30230T0,ICD-10-P-30230T1,ICD-10-P-30230V0,ICD-10-P-30230V1,ICD-10-P-30230W0,ICD-10-P-30230W1,ICD-10-P-30230X0,ICD-10-P-30230X2,ICD-10-P-30230X3,ICD-10-P-30230X4,ICD-10-P-30230Y0,ICD-10-P-30230Y2,ICD-10-P-30230Y3,ICD-10-P-30230Y4,ICD-10-P-30233AZ,ICD-10-P-30233G0,ICD-10-P-30233G2,ICD-10-P-30233G3,ICD-10-P-30233G4,ICD-10-P-30233H0,ICD-10-P-30233H1,ICD-10-P-30233J0,ICD-10-P-30233J1,ICD-10-P-30233K0,ICD-10-P-30233K1,ICD-10-P-30233L0,ICD-10-P-30233L1,ICD-10-P-30233M0,ICD-10-P-30233M1,ICD-10-P-30233N0,ICD-10-P-30233N1,ICD-10-P-30233P0,ICD-10-P-30233P1,ICD-10-P-30233Q0,ICD-10-P-30233Q1,ICD-10-P-30233R0,ICD-10-P-30233R1,ICD-10-P-30233S0,ICD-10-P-30233S1,ICD-10-P-30233T0,ICD-10-P-30233T1,ICD-10-P-30233V0,ICD-10-P-30233V1,ICD-10-P-30233W0,ICD-10-P-30233W1,ICD-10-P-30233X0,ICD-10-P-30233X2,ICD-10-P-30233X3,ICD-10-P-30233X4,ICD-10-P-30233Y0,ICD-10-P-30233Y2,ICD-10-P-30233Y3,ICD-10-P-30233Y4,ICD-10-P-30240AZ,ICD-10-P-30240G0,ICD-10-P-30240G2,ICD-10-P-30240G3,ICD-10-P-30240G4,ICD-10-P-30240H0,ICD-10-P-30240H1,ICD-10-P-30240J0,ICD-10-P-30240J1,ICD-10-P-30240K0,ICD-10-P-30240K1,ICD-10-P-30240L0,ICD-10-P-30240L1,ICD-10-P-30240M0,ICD-10-P-30240M1,ICD-10-P-30240N0,ICD-10-P-30240N1,ICD-10-P-30240P0,ICD-10-P-30240P1,ICD-10-P-30240Q0,ICD-10-P-30240Q1,ICD-10-P-30240R0,ICD-10-P-30240R1,ICD-10-P-30240S0,ICD-10-P-30240S1,ICD-10-P-30240T0,ICD-10-P-30240T1,ICD-10-P-30240V0,ICD-10-P-30240V1,ICD-10-P-30240W0,ICD-10-P-30240W1,ICD-10-P-30240X0,ICD-10-P-30240X2,ICD-10-P-30240X3,ICD-10-P-30240X4,ICD-10-P-30240Y0,ICD-10-P-30240Y2,ICD-10-P-30240Y3,ICD-10-P-30240Y4,ICD-10-P-30243AZ,ICD-10-P-30243G0,ICD-10-P-30243G2,ICD-10-P-30243G3,ICD-10-P-30243G4,ICD-10-P-30243H0,ICD-10-P-30243H1,ICD-10-P-30243J0,ICD-10-P-30243J1,ICD-10-P-30243K0,ICD-10-P-30243K1,ICD-10-P-30243L0,ICD-10-P-30243L1,ICD-10-P-30243M0,ICD-10-P-30243M1,ICD-10-P-30243N0,ICD-10-P-30243N1,ICD-10-P-30243P0,ICD-10-P-30243P1,ICD-10-P-30243Q0,ICD-10-P-30243Q1,ICD-10-P-30243R0,ICD-10-P-30243R1,ICD-10-P-30243S0,ICD-10-P-30243S1,ICD-10-P-30243T0,ICD-10-P-30243T1,ICD-10-P-30243V0,ICD-10-P-30243V1,ICD-10-P-30243W0,ICD-10-P-30243W1,ICD-10-P-30243X0,ICD-10-P-30243X2,ICD-10-P-30243X3,ICD-10-P-30243X4,ICD-10-P-30243Y0,ICD-10-P-30243Y2,ICD-10-P-30243Y3,ICD-10-P-30243Y4,ICD-10-P-30250G0,ICD-10-P-30250G1,ICD-10-P-30250H0,ICD-10-P-30250H1,ICD-10-P-30250J0,ICD-10-P-30250J1,ICD-10-P-30250K0,ICD-10-P-30250K1,ICD-10-P-30250L0,ICD-10-P-30250L1,ICD-10-P-30250M0,ICD-10-P-30250M1,ICD-10-P-30250N0,ICD-10-P-30250N1,ICD-10-P-30250P0,ICD-10-P-30250P1,ICD-10-P-30250Q0,ICD-10-P-30250Q1,ICD-10-P-30250R0,ICD-10-P-30250R1,ICD-10-P-30250S0,ICD-10-P-30250S1,ICD-10-P-30250T0,ICD-10-P-30250T1,ICD-10-P-30250V0,ICD-10-P-30250V1,ICD-10-P-30250W0,ICD-10-P-30250W1,ICD-10-P-30250X0,ICD-10-P-30250X1,ICD-10-P-30250Y0,ICD-10-P-30250Y1,ICD-10-P-30253G0,ICD-10-P-30253G1,ICD-10-P-30253H0,ICD-10-P-30253H1,ICD-10-P-30253J0,ICD-10-P-30253J1,ICD-10-P-30253K0,ICD-10-P-30253K1,ICD-10-P-30253L0,ICD-10-P-30253L1,ICD-10-P-30253M0,ICD-10-P-30253M1,ICD-10-P-30253N0,ICD-10-P-30253N1,ICD-10-P-30253P0,ICD-10-P-30253P1,ICD-10-P-30253Q0,ICD-10-P-30253Q1,ICD-10-P-30253R0,ICD-10-P-30253R1,ICD-10-P-30253S0,ICD-10-P-30253S1,ICD-10-P-30253T0,ICD-10-P-30253T1,ICD-10-P-30253V0,ICD-10-P-30253V1,ICD-10-P-30253W0,ICD-10-P-30253W1,ICD-10-P-30253X0,ICD-10-P-30253X1,ICD-10-P-30253Y0,ICD-10-P-30253Y1,ICD-10-P-30260G0,ICD-10-P-30260G1,ICD-10-P-30260H0,ICD-10-P-30260H1,ICD-10-P-30260J0,ICD-10-P-30260J1,ICD-10-P-30260K0,ICD-10-P-30260K1,ICD-10-P-30260L0,ICD-10-P-30260L1,ICD-10-P-30260M0,ICD-10-P-30260M1,ICD-10-P-30260N0,ICD-10-P-30260N1,ICD-10-P-30260P0,ICD-10-P-30260P1,ICD-10-P-30260Q0,ICD-10-P-30260Q1,ICD-10-P-30260R0,ICD-10-P-30260R1,ICD-10-P-30260S0,ICD-10-P-30260S1,ICD-10-P-30260T0,ICD-10-P-30260T1,ICD-10-P-30260V0,ICD-10-P-30260V1,ICD-10-P-30260W0,ICD-10-P-30260W1,ICD-10-P-30260X0,ICD-10-P-30260X1,ICD-10-P-30260Y0,ICD-10-P-30260Y1,ICD-10-P-30263G0,ICD-10-P-30263G1,ICD-10-P-30263H0,ICD-10-P-30263H1,ICD-10-P-30263J0,ICD-10-P-30263J1,ICD-10-P-30263K0,ICD-10-P-30263K1,ICD-10-P-30263L0,ICD-10-P-30263L1,ICD-10-P-30263M0,ICD-10-P-30263M1,ICD-10-P-30263N0,ICD-10-P-30263N1,ICD-10-P-30263P0,ICD-10-P-30263P1,ICD-10-P-30263Q0,ICD-10-P-30263Q1,ICD-10-P-30263R0,ICD-10-P-30263R1,ICD-10-P-30263S0,ICD-10-P-30263S1,ICD-10-P-30263T0,ICD-10-P-30263T1,ICD-10-P-30263V0,ICD-10-P-30263V1,ICD-10-P-30263W0,ICD-10-P-30263W1,ICD-10-P-30263X0,ICD-10-P-30263X1,ICD-10-P-30263Y0,ICD-10-P-30263Y1,ICD-10-P-30273H1,ICD-10-P-30273J1,ICD-10-P-30273K1,ICD-10-P-30273L1,ICD-10-P-30273M1,ICD-10-P-30273N1,ICD-10-P-30273P1,ICD-10-P-30273Q1,ICD-10-P-30273R1,ICD-10-P-30273S1,ICD-10-P-30273T1,ICD-10-P-30273V1,ICD-10-P-30273W1,ICD-10-P-30277H1,ICD-10-P-30277J1,ICD-10-P-30277K1,ICD-10-P-30277L1,ICD-10-P-30277M1,ICD-10-P-30277N1,ICD-10-P-30277P1,ICD-10-P-30277Q1,ICD-10-P-30277R1,ICD-10-P-30277S1,ICD-10-P-30277T1,ICD-10-P-30277V1,ICD-10-P-30277W1,ICD-10-P-30280B1,ICD-10-P-30283B1

Wound Complication

ICD-9-D-99883, ICD-9-D-99832, ICD-9-D-99830, ICD-10-D-T8131XA, ICD-10-D-T8131XD, ICD-10-D-T8131XS, ICD-10-D-A4852, ICD-10-D-B871
